# Medical costs of Alpha-1 antitrypsin deficiency-associated COPD in the United States

**DOI:** 10.1186/s13023-020-01523-4

**Published:** 2020-09-23

**Authors:** Jan Sieluk, Julia F. Slejko, Henry Silverman, Eleanor Perfetto, C. Daniel Mullins

**Affiliations:** 1 Pharmaceutical Health Services Research Department, University of Maryland, School of Pharmacy, 220 Arch Street, Baltimore, MD 21201 USA; 2 OptumLabs Visiting Fellow, OptumLabs, Cambridge, MA USA; 3 University of Maryland School of Medicine, Baltimore, MD USA; 4 National Health Council, Washington, DC USA

**Keywords:** Alpha-1 antitrypsin deficiency, Rare disease, Chronic obstructive pulmonary disease, Cost-of-illness, Economic analysis, Healthcare resource use

## Abstract

**Background:**

There are limited data on economic aspects of the genetic variant of chronic obstructive pulmonary disease (COPD) in the context of the more prevalent form of COPD. The objective of this study was to isolate the healthcare resource utilization and economic burden attributable to the presence of a genetic factor among COPD patients with and without Alpha-1 Antitrypsin Deficiency (AATD), twelve months before and after their initial COPD diagnosis.

**Methods:**

Retrospective analysis of OptumLabs® Data Warehouse claims (OLDW; 2000–2017). The OLDW is a comprehensive, longitudinal real-world data asset with de-identified lives across claims and clinical information. AATD-associated COPD cases were matched with up to 10 unique non-AATD-associated COPD controls. Healthcare resource use and costs were assigned into the following categories: office (OV), outpatient (OP), and emergency room visits (ER), inpatients stays (IP), prescription drugs (RX), and other services (OTH). A generalized linear model was used to estimate total pre- and post-index (initial COPD diagnosis) costs from a third-party payer’s perspective (2018 USD) controlling for confounders. Healthcare resource utilization was estimated using a negative binomial regression.

**Results:**

The study population consisted of 8881 patients (953 cases matched with 7928 controls). The AATD-associated COPD cohort had higher expenditures and use of office visits (OV) and other (OTH) services, as well as OV, outpatient (OP), emergency room (ER), and prescription drugs (RX) before and after the index date, respectively. Adjusted total all-healthcare cost ratios for AATD-associated COPD patients as compared to controls were 2.04 [95% CI: 1.60–2.59] and 1.98 [95% CI: 1.55–2.52] while the incremental cost difference totaled $6861 [95% CI: $3025 - $10,698] and $5772 [95% CI: $1940 - $9604] per patient before and after the index date, respectively.

**Conclusions:**

Twelve months before and after their initial COPD diagnosis, patients with AATD incur higher healthcare utilization costs that are double the cost of similar COPD patients without AATD. This study also suggests that increased costs of AATD-associated COPD are not solely attributable to augmentation therapy use. Future studies should further explore the relationship between augmentation therapy, healthcare resource use, and other AATD-associated COPD expenditures.

## Background

Genomic medicine implies that diseases with genetic components may require a different approach to diagnosis, management, and treatment, as compared to diseases without known inheritable components [1]. For instance, patients with HER2-positive breast cancer are managed differently as compared to women with triple-negative disease [2, 3]; patients with MYH-associated polyposis require thorough medical scrutiny due to an almost 100% risk of developing colorectal cancer before reaching the age of 65 [4, 5]; patients with Alpha-1 Antitrypsin Deficiency who developed chronic obstructive pulmonary disease (AATD-associated COPD) may need augmentation therapy, not indicated for COPD patients with normal serum levels of Alpha-1 Antitrypsin protein (AAT) [6–14].

Given the significant morbidity and mortality of COPD in the United States and worldwide, coupled with an increasing recognition and economic burden of the disease, it is surprising how little is known in terms of the contemporary direct medical costs of AATD-associated COPD [6, 15–20]. The World Health Organization estimated that about 5% of all deaths worldwide are attributable to COPD [6, 15]. In the United States, there are about 10 million adult patients with COPD, resulting in significant morbidity, mortality, and costs [16, 17, 19]. Based on advancements in genomics, a genetic factor was discovered, which can play a significant role in the pathomechanism of COPD among a subgroup of AATD-affected patients [21, 22]. AATD is an autosomal, co-dominant condition that most commonly affects lungs, causing a number of health-related problems [23]. AATD is associated with the development of an early emphysema and chronic bronchitis, which may be collectively described as COPD; less frequently associated with cirrhosis (in patients over 50 years of age and among infants – fulminant neonatal hepatic syndrome), hepatocellular carcinoma, vasculitis and rarely skin diseases like necrotizing panniculitis [21, 24–27].

Previously, the predominant method for conducting a rare-disease cost study was through primary data collection. Today, however, a large enough sample from a claims database belonging to one of the largest insurers in the United States offers an opportunity to study AATD [28]. In a broader perspective, the size and availability of insurance datasets in the United States opens a new chapter to study rare diseases, diseases with genetic components, as well as orphan drugs uses. Historically, the sample size has been a major barrier to meaningful statistical inference [29–33]. The authors embarked on this research endeavor to address an existing gap in the literature, by conducting a rigorous estimation of health care resource utilization and costs of the genetic variant of COPD (AATD-associated COPD) in the context of the more prevalent, nongenetic form of COPD, twelve months before and after an initial COPD diagnosis claim.

## Methods

### Data source

The study involves a retrospective analysis of claims data from the OptumLabs® Data Warehouse (OLDW), which includes de-identified claims data for privately-insured and Medicare Advantage enrollees in a large, private, U.S. health plan. The database contains longitudinal health information on enrollees, representing a diverse mixture of ages, ethnicities and geographical regions across the United States. The health plan provides comprehensive full insurance coverage for physician, hospital, and prescription drug services [34].

All available healthcare costs were measured and adjusted for inflation to 2018 USD, using the medical care component of the Consumer Price Index. Cost data represent amounts paid by the health plan, and was operationalized as a sum of paid expenditures covered by the Data were analyzed using Stata 14/MP (StataCorp, TX, USA). Institutional Review Board (IRB) approval was obtained from the IRB of the University of Maryland, Baltimore, on July 27, 2016 (HP-00068329).

### Study design and variables

**Fig. 1:**
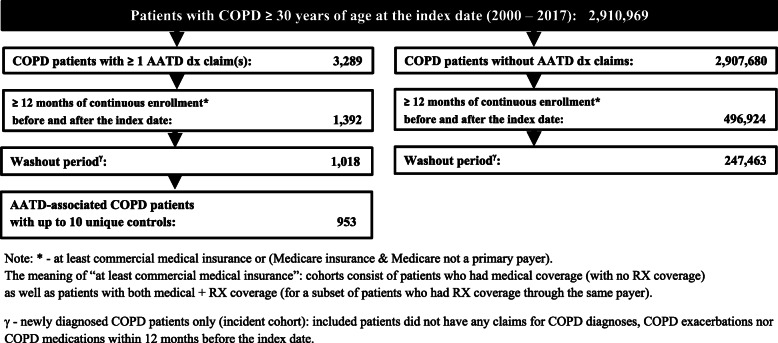
Cohort identification flowchart

AATD-associated COPD patients were identified based on ≥1 diagnosis claim(s) for AATD (ICD-9: 273.4; ICD-10: E88.01) and ≥ 2 diagnosis claims for COPD (emphysema: ICD-9: 492.x, ICD-10: J43.x; chronic bronchitis: ICD-9: 491.x, ICD-10: J40, J41.x, J42.x; bronchiectasis: ICD-9: 494.x, ICD-10: J47; chronic airway obstruction, not elsewhere classified: ICD-9: 496, ICD-10: J44). After AATD-associated COPD patients were excluded from the COPD patient pool, non-Alpha-1-associated COPD cohort was identified based on ≥2 diagnosis claims for COPD. The index date was the date of the initial COPD diagnosis claim. Only adult patients at least 30 years of age at the index date were included in the analysis. The time horizon for this study was January 2000 – August 2017. Patients over 65 years of age for whom Medicare was their primary payer were excludedto avoid bias resulting from unobserved costs. At least 12 months of continuous enrollment before and after the index date was required for both cohorts (Fig. 1). The cohorts include patients who had medical coverage (with no RX coverage) as well as patients with both medical and RX coverage (for a subset of patients who had RX coverage through the same payer). Only newly diagnosed patients (incident cohort) who did not have COPD diagnoses nor pharmacy claims for COPD medications (identified using National Drug Codes, NDC) 12 months before the index date denoting the washout period were included in the analysis.

Patient demographics and clinical characteristics collected from enrollment and claims files at the index date included: age, age categories, gender, race/ethnicity, census region and Quan’s Charlson Comorbidity Index (containing both ICD-9-CM and ICD-10-CM diagnoses codes) calculated within 365 days preceding the index date (including index date) [35–37]. Missing race/ethnicity was imputed using multinomial logistic regression.

Insurers’ costs were calculated and categorized into the following resource categories: all costs (TOT), office visits (OV), outpatient visits (office visits in a hospital setting; OP), emergency room visits (ER), inpatients stays (IP), prescription drugs costs (RX), and other costs (OTH). Costs were defined as amounts paid by insurers’ (third-party payers’ perspective) and measured as all healthcare costs, not COPD-only costs.

AATD-associated COPD patients with ≥1 diagnosis claim(s) for AATD were matched with up to 10 unique non-AATD-associated COPD controls. The exact matching algorithm without replacement included the following variables: gender, Quan’s Charlson Comorbidity Score, census region, race/ethnicity, age at diagnosis (+/− 1 year), year of COPD diagnosis and oxygen use (Yes/No; within the first 12 months after the index date) as a proxy for COPD severity.

### Multivariate analyses

There were two hypotheses: 1): Patients with AATD utilize more health care resources (incurring higher direct medical costs) as compared to patients without AATD within 12 months before receiving their initial COPD diagnosis. 2) Patients with AATD-associated COPD utilize more health care resources (incurring higher direct medical costs) as compared to patients with non-AATD-associated COPD within 12 months before and after receiving their initial COPD diagnosis.

The sum of ER, OV, and OP visits, RX, IP and OTH per patient were modeled in pre- and post-index periods separately using models for count data. Overall, four different count models have been tested: a Poisson, a zero-inflated Poisson, a negative binomial, and zero-inflated negative binomial models [38].

The use of a particular count data model was based on the goodness-of-fit criteria. The goodness-of-fit criteria for four models included comparisons of the Akaike’s (AIC) and Bayesian Information Criterion (BIC) for every resource category separately, both before and after the index date [38].

To test the hypotheses linked to resource utilization among AATD-associated COPD patients, a dummy variable on the Alpha-1 status for each healthcare resource category before and after index date was investigated for statistical significance (α = 0.05).

Total 12-month pre- and post-index costs were modeled using a generalized linear model (GLM). In addition, the 12-month pre-and post-index date costs were modeled separately and reported for each resource category.

The choice of the link function and distribution family was conducted sequentially [38]. Six different GLMs were compared based on different links and family distributions: log-gamma, square root – gamma, log – Gaussian, square root – Gaussian, log-Poisson, and square-root Poisson model [38].

The link test was conducted using the Box-Cox approach, the Pearson Correlation test, the Pregibon link test and the modified Hosmer and Lemeshow test [38].

The family distribution was assessed using the modified Park test [38]. This test investigates the relationship between the variance of the error term and mean, and the value of the coefficient serves as the guidance for the choice of family distribution [38]. Misspecifications test for GLMs were conducted using the RESET test [39].

The choice of a final model was based on the goodness-of-fit criteria (the lowest AIC and BIC values) across tested GLM models, as well as the assessment of deviance residuals and a Q-Q plot of deviance residuals.

Adjusted results are presented as ratios of expected costs between both cohorts as well as grand margins. To test the hypotheses, in the GLM model, dummy variable on the Alpha-1 status was investigated for statistical significance (α = 0.05) and then bootstrapped with replacement 1000 times.

To assess whether a two-stage (hurdle) model should be used, the proportions of patients with total pre- and post-index costs equal to zero were calculated.

### Sensitivity analyses

In the one-way sensitivity analysis, the impact of the number of AATD diagnosis claims on costs was investigated: the total cost estimates were re-calculated for AATD-associated COPD patients with ≥2 diagnosis claims for AATD to account for the potential of rule-out diagnoses (testing for AATD). In addition, the impact of the 1:10 matching ratio on total pre- and post-index costs was investigated.

In a multi-way sensitivity analysis, both the impact of ≥2 AATD diagnosis claims and the 1:10 matching ratio on the total pre-and post-index costs were investigated at the same time.

## Results

### Population characteristics and matching

A cohort identification flowchart is presented in Fig. 1. Overall, out of 1018 AATD-associated COPD patients who met inclusion criteria, 953 cases had up to 10 unique matched controls by randomly matching with 7928 patients non-AATD-associated COPD patients.

Table 1 lists the demographic and clinical characteristics of the analyzed cohorts before matching. Patients with AATD-associated COPD were statistically different in terms of all baseline covariates except for mean age and Charlson Comorbidity Index (Table 1). Table A in the Appendix documents the distribution of demographic and clinical characteristics of study cohorts after matching.

**Table 1:** Demographic and clinical differences among study cohorts with RX coverage

	AATD-COPD Cohort (cases)		non-AATD-COPD Cohort (controls)		***P*** value^**c**^
	***N*** = 749		***N*** = 5,391		
	No. of patients	(Column %) / [median]	No. of patients	(Column %) / [median]	
**Age,** ***mean (SD)***	57.10 (11.84)	[57]	52.13 (8.45)	[53]	< 0.001
**Age categories,** ***n (%)***					< 0.001
*30 - 39*	51	(7)	453	(8)	
*40 - 49*	151	*(20)*	1,445	*(27)*	
*50 - 59*	242	*(32)*	2,382	*(44)*	
*60 - 64*	107	*(14)*	885	*(16)*	
*>=65*	198	*(26)*	226	*(4)*	
**Gender,** ***n (%)***					0.361
*Male*	345	*(46)*	2,579	*(48)*	
*Female*	404	*(54)*	2,812	*(52)*	
**Race/Ethnicity**^**a**^**,** ***n (%)***					0.146
*White*	625	*(83)*	4,607	*(85)*	
*African American*	42	*(6)*	238	*(4)*	
*Hispanic*	32	*(4)*	276	*(5)*	
*Unknown*	45	*(6)*	242	*(4)*	
					0.014
**Census region,** ***n (%)***					
*Northeast*	89	*(12)*	475	(9)	
*Midwest*	241	*(32)*	1,756	(33)	
*South*	304	*(41)*	2,418	(45)	
*West*	115	*(15)*	742	(14)	
**Charlson Comorbidity Score**^**a**^**,** ***n (%)***					< 0.001
*1*	433	*(58)*	3,680	*(68)*	
*2*	125	*(17)*	783	*(15)*	
*3*	76	*(10)*	470	*(9)*	
4	42	*(6)*	191	*(4)*	
≥5	73	*(10)*	267	*(5)*	

^a^Missing race/ethnicity was replaced using multinomial logistic regression

^b^Quan's Enhanced Charlson Comorbidity Score calculated within 365 days before (including) the index

^c^Calculated with the use of two-sample Mann-Whitney Test for mean age, and chi-square test for categorical variables

Among 953 AATD-associated COPD cases, 114 (12%) patients were diagnosed with AATD before the first COPD diagnosis claim, 37 (4%) patients were diagnosed with AATD and COPD on the same day, and 802 (84%) were diagnosed with AATD after the first COPD diagnosis date. Table 2 presents the temporal relationship between the first AATD and COPD diagnoses dates among AATD-associated COPD patients, stratified by time horizons analyzed in this study.

**Table 2:** Timing between AATD and COPD diagnoses stratified by time period

AATD-associated COPD subgroup	AATD-COPD Cohort			
	***N*** = 953			
	Mean difference (years)	SD	Median	Range
**AATD diagnosis before COPD diagnosis (N = 114; 12%)**^**a**^				
*2000 - 2009*	1.17	1.12	0.9	4.27
*2010 - 2017*	1.77	1.87	1.33	9.75
**AATD diagnosis after COPD diagnosis (N = 802; 84%)**^**a**^				
*2000 - 2009*	5.42	3.82	4.84	17.38
*2010 - 2017*	2.39	1.8	1.62	7.67

^a^time horizons represent the date of the first COPD diagnosis

### Healthcare resource utilization: multivariate analyses on matched cohorts

The Poisson model did not offer a good fit for any of the healthcare resource categories; hence, a negative binomial model was used as it offered the best fit across all four models tested.

The adjusted effects of Alpha-1 Antitrypsin Deficiency on health care resource utilization before and after the index date are presented in Table 3.

**Table 3:** Adjusted healthcare resource utilization before and after the index date

	Before index date			After index date		
Healthcare resource	Attributable effect^υ^	95% CI (ROBUST)	*P* value	Attributable effect^υ^	95% CI (ROBUST)	*P* value
Office visits	**+ 0.918**	**0.207 - 1.628**	**0.011**	**+ 1.712**	**0.897 - 2.528**	**< 0.001**
Outpatient visits	+ 0.209	-0.047 - 0.465	0.110	**+ 0.951**	**0.501 - 1.401**	**< 0.001**
ER visits	- 0.015	-0.072 - 0.042	0.602	**+ 0.089**	**0.013 - 0.165**	**0.021**
Inpatient days	- 0.007	-0.349 - 0.335	0.968	+ 0.017	-0.606 - 0.640	0.957
Other services	**+ 0.879**	**0.406 - 1.351**	**< 0.001**	**+ 2.171**	**1.463 - 2.878**	**< 0.001**
Prescription drugs^ω^	+ 0.050	-0.885 - 0.985	0.100	+ 0.449	-8.904 - 2.895	0.482

κ: fully adjusted incidence rate ratio attributable to the presence of AATD; negative binomial model adjusted for age category, gender, race, period of COPD diagnosis census region, oxygen use, Charlson Comorbidity Score, and interaction terms: AATD*CCI, AATD*Oxygen use and AATD*Period of COPD diagnosis

ϒ: number of additional visits/Inpatient days/Prescription drugs on a raw scale attributable to the presence of AATD; negative binomial model

Ω: for a subset of patients who had prescription drug coverage through the same payer

Comparing the adjusted healthcare resource use across both COPD samples, the AATD-associated COPD cohort utilized more healthcare services across OV and OTH resource categories before the index date, as well as more OV, OP, ER, and OTH healthcare services after the index date (Table 3).

### Incremental costs

The GLM model with log link and gamma distribution offered the best model fit for overall healthcare costs and all resource categories costs before and after the index date. A two-stage modeling approach was not used as the number of patients with total pre- and post-index costs equal to zero were much less than 5% among both cases and cohorts. Spending stratified by healthcare resource categories with respect to the index date is presented in Table 4. Overall, the AATD-associated COPD cohort incurred 2.036 (95% CI: 1.601–2.590) times and 1.976 (95% CI: 1.550–2.517) times the total 12-month cost incurred by the non-Alpha-1 COPD cohort before and after the index date, respectively.

**Table 4:** Adjusted cost ratios before and after the index date

	Before index date			After index date		
Healthcare resource	Cost ratio^ε^	95% CI (ROBUST)	*P* value	Cost Ratio^ε^	95% CI (ROBUST)	*P* value
Office visits	1.195	0.939 - 1.519	0.147	1.210	0.929 - 1.575	0.157
Outpatient visits	0.855	0.685 - 1.067	0.164	**1.490**	**1.055 - 2.103**	**0.024**
ER visits	0.844	0.627 - 1.136	0.263	1.160	0.830 - 1.624	0.386
Inpatient days	0.844	0.531 - 1.342	0.473	0.906	0.568 - 1.444	0.677
Other services	**6.245**	**3.424 - 11.388**	**< 0.001**	**11.834**	**7.834 - 17.875**	**< 0.001**
Prescription drugs^ω^	0.995	0.782 - 1.267	0.970	**1.372**	**1.087 - 1.731**	**0.008**

ε: Fully adjusted cost ratio; generalized Linear Model (gamma family, log link) adjusted for age category, gender, race, period of COPD diagnosis census region, oxygen use, Charlson Comorbidity Score, and interaction terms: AATD*CCI, AATD*Oxygen use and AATD*Period of COPD diagnosis

ϒ: number of additional visits/IP days/Prescription drugs on a raw scale attributable to the presence of AATD; generalized linear model

Ω: for a subset of patients who had prescription drug coverage through the same payer

Only OTH services used by AATD-associated COPD cohort were more expensive (cost ratio: 6.245; 95% CI: 3.424–11.388) before the index date, resulting in an additional $1560 (95% CI: $739 – $2454; data not shown).

After the index date, the cases incurred higher OP costs (cost ratio: 1.490; 95% CI: 1.055–2.103), higher OTH costs (cost ratio: 11.834; 95% CI: 7.834–17.875) and RX costs (cost ratio: 1.372; 95% CI: 1.087–1.731; Table 4), resulting in an additional $5782 (95% CI: $3982 - $7582) and $1974 (95% CI: $141 - $3447) in OTH and RX expenditures, respectively.

### Sensitivity analysis

In the sensitivity analysis, the 12-month total cost estimates were re-calculated for AATD-associated COPD patients with ≥2 AATD diagnosis claims to account for the possibility of rule-out diagnoses (testing for AATD).

Of note, the 12-month total cost ratio for AATD-associated COPD patients with ≥2 diagnosis claims was even higher and totaled 2.768 (95% CI: 2.087–3.672) resulting in an additional $14,753 (95% CI: $8909 – $20.598) incurred before the index date. After the index date, the cost ratio equaled 2.668 (95% CI: 2.002–3.554) resulting in an additional $13,029 (95% CI: $7232 – $18,825) spent.

When analyzing cases with 10 unique matches only, the cost ratio was very close to the base-case estimate and totaled 2.035 (95% CI: 1.541–2.686) resulting in an additional $8486 (95% CI: 3690 – 13,282) paid by insurers’ before the index date. After the index date, the cost ratio equaled 1.993 (95% CI: 1.508–2.636) resulting in an additional $7361 (95% CI: $2592 – $12,130) in costs.

In the multi-way sensitivity analysis, the impact of both ≥2 diagnosis claims for AATD and 10 unique matches was investigated. Before the index date, the cost ratio was higher than in the base-case analysis and was equal to 2.873 (95% CI: 2.078–3.971), resulting in an additional $17,190 ($8965 – $25,415) incurred by insurers. After the index date, the cost ratio was equal to 2.796 (95% CI: 2.017–3.877), which resulted in an additional $15,278 (95% CI: $7123 – $23,432).

## Discussion

Patients, clinicians, and policy-makers agree that there is no such thing as “one size fits all” medicine. This is particularly true when the causes of a disease and the best treatment approach are related to biology and genomics [1]. Patients with rare forms of diseases often use tailored treatments. Unfortunately, the rare forms of disease are often associated with treatments that are more expensive. Therefore, patients with rare genetic diseases may have higher healthcare costs and may need to see more specialists.

Nevertheless, even without underlying AATD, COPD has been described as a disease with a high and increasing economic burden over time and substantial incremental costs as compared to patients without COPD [28, 40].

Where prior cost studies conducted in the United States merely estimated the total cost of AATD care, this study delineated the cost difference between treating AATD- associated COPD from the “garden variety” form of COPD [28]. Until today, despite abundant COPD cost-of-illness research, there has been no understanding of contemporary healthcare resource utilization and costs of the genetic variant of COPD in the United States. In a broader perspective, understanding the proportion of economic burden of a rare disease that is attributable to genetic factors is especially important given that about 80% of rare diseases have genetic causes [41].

This study reveals that patients with AATD-associated COPD have nearly twice the all-healthcare costs of similar patients without AATD twelve months before and after their initial COPD diagnosis claim. While AATD-associated COPD patients are usually younger as compared to non-AATD-associated COPD, the observed age difference in this study was reduced due to matching. Among patients who have been diagnosed with AATD prior to COPD diagnosis, the results may suggest that: 1) individuals were identified because of family testing because an immediate relative was diagnosed with AATD; or 2) individuals were identified with liver disease due to AATD. Both groups might have increased medical expenses because, in the first scenario, careful follow-up with routine visits for pulmonary and liver function testing is recommended for health AATD individuals. In the second case, there can be significant expenses for those with liver disease due to AATD. However, in this research, the authors did not investigate healthcare resource use and costs among AATD patients with liver disease (including liver transplants) separately.

In previous U.S.-based research, it was found that the mean annual cost estimates ranged between $20,673 and $30,948 per patient, depending on the phenotype [42]. The mean yearly cost for patients receiving α1-antiprotease was $40,123 (median $36,000) in 2001 when there was only one augmentation product available in the U.S. market [42]. In 2003 and 2004, two additional α1-antiprotease products were introduced; following the two new entrants, costs of augmentation therapy rose to $115,218/year (95% CI: $63,676 - $166,760; data not shown).

A recent European study found no difference between augmentation users and non-users in terms of Health-Related Quality of Life, with costs of augmentation therapy equal to €72,000 (approximately $79,575) in 2017 [43]. In addition, AATD-associated COPD patients in Germany incurred somewhat lower costs as compared to similar patients without AATD; the European researchers adjusted the analyses for variables unavailable in our study, and significant differences in patient definitions or financing mechanisms of the healthcare systems in the U.S. and Europe have to be acknowledged [44, 45].The potential for confounding and applicable limitations to this research warrant an additional comment. First, recognized experts in regression-based COI have noted that the use of regression can be problematic in case of confounding by lifestyle choices, i.e. smoking.

Moreover, a potential limitation could pertain to the prevalence and the severity of under-diagnosing of AATD. One study reported that up 10% of COPD patients may be A1AT deficient [8].

If true, this is especially important in this analysis, as the comparisons between AATD-associated COPD and non-AATD-associated COPD patients would be potentially underestimated when matching with similar controls. On the contrary, AATD patients with milder forms of the disease, or those with AATD who did not yet develop COPD might incur lower costs. Those populations were not within the scope of this analysis. While certain demographic variables, like race/ethnicity or geographic location, were adjusted for in the analyses, some environmental factors, patient’s characteristics (AATD phenotype), enabling resources and personal health practices cannot be accounted for with the use of administrative claims database. In other words, insurance companies do not routinely collect data on, for example, health beliefs or social relationships with other patients affected by a particular disease. Such patient-centered analyses could possibly constitute future research on AATD with the use of patient-reported measures based on patient registries linked to claims datasets.

As well, the list of available matching variables is subject to the common limitations of administrative claims databases and does not include some key confounders like socioeconomic status, AATD-associated COPD disease severity expressed as the GOLD stage, clinical confirmation of disease, FEV1 values or the Tiffenau-Pinelli index. Patients with more severe forms of COPD might incur higher costs both before and after COPD diagnosis. Hence, some caution needs to be exercised while interpreting the results of this study.

It was also found that the AATD-associated COPD patients used more RX after their initial COPD diagnosis claim. However, the AATD-associated COPD patients are routinely excluded from clinical trials of COPD medications [27]. There are several other limitations. One of the issues is the lack of AATD phenotype (genetic) information in administrative claims databases. With over 100 phenotypes described to date, there are likely subgroups of AATD patients who present with a more severe form of COPD as compared to their counterparts, some of them might also incur additional costs due to liver disease. Such costs could be further exacerbated by the unavailability of the smoking status nor a proxy such as smoking pack-years. Therefore, AATD patients with severe phenotypes would be hardly comparable to patients without AATD. In addition, factors prompting the initial COPD diagnosis claim among patients affected and unaffected by AATD might differ in an unknown manner.

## Conclusions

AATD-associated COPD patients have two-fold higher costs than non-AATD-associated COPD patients before and years after initial COPD diagnosis. These findings should be of high priority and interest to payers because their spending on the genetic variant of COPD has been on the rise for the past decade and is expected to increase further [20, 40, 46, 47]. This could motivate payers to invest in AATD-COPD disease detection and management endeavors. The higher use of ER visits and prescription medications may be mitigated if AATD-associated COPD patients enrolled in a disease management program [48, 49]. For example, among AATD-affected patients, the Alpha-1 Disease Management and Prevention Program (ADMAPP) has been demonstrated to reduce the number of outpatient and ER visits, as well as exacerbations, due to, but not limited to, optimization in the use and improved adherence to prescription medicines for COPD [48]. Alternatively or in collaboration, pharmaceutical manufacturers could sponsor a disease-management program with the intent to increase medication adherence, reduce costs and improve quality of care among COPD-affected patients [50].

This analysis detected significant delays in getting an AATD diagnosis after the initial COPD diagnosis [51–53]. This finding is important to patients and payers, as a timely diagnosis of AATD status would facilitate more personalized care, through a reduction in the number of unscheduled physician visits [48]. Building upon the premise that enrollment into a disease management program could potentially mitigate excess healthcare expenditures, earlier detection may lead to earlier benefit from ADMAPP. Insurers should use this information to undertake a CEA study, as well as a budget impact analysis of participating in an AATD-associated COPD disease management program (standard of care: COPD management program) to help address more directly the value of timely AATD diagnosis and management. Such analyses could add additional information on the true impact and the value of reducing the diagnostic delay, as well as regarding the potential of tailored disease-management programs and pharmacological management of COPD to improve outcomes and reduce health care costs in this unique population.

## Data Availability

The data that support the findings of this study are available from the OptumLabs® Data Warehouse (OLDW) but restrictions apply to the availability of these data, which were used under license for the current study, and so are not publicly available. Data are however available from the authors upon reasonable request and with permission of the OLDW.

## Appendix

**Table 5:** Demographic and clinical characteristics of study cohorts after matching

	AATD-COPD COHORT (CASES)		NON-AATD-COPD COHORT (CONTROLS)	
	***N*** = 953		***N*** = 7,928	
	No. of patients	(Column %) / [median]	No. of patients	(Column %) / [median]
**Age,** ***mean (SD)***	56.24 (10.74)	[56]	55.17 (9.62)	[56]
**Age categories,** ***n (%)***				
*30 - 39*	57	(6)	445	(6)
*40 - 49*	191	*(20)*	1,697	*(20)*
*50 - 59*	355	*(37)*	3,194	*(37)*
*60 - 64*	174	*(18)*	1,537	*(18)*
*>=65*	176	*(18)*	1,055	*(18)*
**Gender,** ***n (%)***				
*Male*	456	*(48)*	3,812	*(48)*
*Female*	497	*(52)*	4,116	*(52)*
**Race/Ethnicity**^**a**^**,** ***n (%)***				
*White*	877	*(92)*	7,526	*(95)*
*African American*	39	*(4)*	201	*(3)*
*Hispanic*	33	*(3)*	195	*(2)*
**Census region,** ***n (%)***				
*Northeast*	104	*(11)*	104	*(11)*
*Midwest*	287	*(30)*	287	*(30)*
*South*	414	*(43)*	414	*(43)*
*West*	148	*(16)*	148	*(16)*
**Charlson Comorbidity Score**^**a**^**,** ***n (%)***				
*1*	596	*(63)*	5,416	*(68)*
*2*	161	*(17)*	1,282	*(16)*
*3*	90	*(9)*	592	*(7)*
4	40	*(4)*	207	*(3)*
≥5	66	*(7)*	431	*(5)*

^a^Missing race/ethnicity was replaced using multinomial logistic regression

^b^Quan's Enhanced Charlson Comorbidity Score calculated within 365 days before (including) the index date

**Table 6:** Demographic and clinical characteristics of study cohorts with prescription drug coverage after matching

	AATD-COPD COHORT (CASES)		NON-AATD-COPD COHORT (CONTROLS)	
	***N*** = 953		***N*** = 7,928	
	No. of patients	(Column %) / [median]	No. of patients	(Column %) / [median]
**Age,** ***mean (SD)***	56.24 (10.74)	[56]	55.17 (9.62)	[56]
**Age categories,** ***n (%)***				
*30 - 39*	57	(6)	445	(6)
*40 - 49*	191	*(20)*	1,697	*(20)*
*50 - 59*	355	*(37)*	3,194	*(37)*
*60 - 64*	174	*(18)*	1,537	*(18)*
*>=65*	176	*(18)*	1,055	*(18)*
**Gender,** ***n (%)***				
*Male*	456	*(48)*	3,812	*(48)*
*Female*	497	*(52)*	4,116	*(52)*
**Race/Ethnicity**^**a**^**,** ***n (%)***				
*White*	877	*(92)*	7,526	*(95)*
*African American*	39	*(4)*	201	*(3)*
*Hispanic*	33	*(3)*	195	*(2)*
**Census region,** ***n (%)***				
*Northeast*	104	*(11)*	104	*(11)*
*Midwest*	287	*(30)*	287	*(30)*
*South*	414	*(43)*	414	*(43)*
*West*	148	*(16)*	148	*(16)*
**Charlson Comorbidity Score**^**a**^**,** ***n (%)***				
*1*	596	*(63)*	5,416	*(68)*
*2*	161	*(17)*	1,282	*(16)*
*3*	90	*(9)*	592	*(7)*
4	40	*(4)*	207	*(3)*
≥5	66	*(7)*	431	*(5)*

^a^Missing race/ethnicity was replaced using multinomial logistic regression

^b^Quan's Enhanced Charlson Comorbidity Score calculated within 365 days before (including) the index date

## Abbreviations

AAT: Alpha-1 antitrypsin protein (AAT)
AATD: Alpha-1 antitrypsin deficiency
ADMAPP: the Alpha-1 Disease Management and Prevention Program
AIC: Akaike’s Information Criterion
BIC: Bayesian Information Criterion
COPD: Chronic obstructive pulmonary disease
ER: Emergency room
GLM: Generalized linear model
HER2: Human epidermal growth factor *receptor* 2 (*HER2*)
IP: Inpatient
NDC: National drug codes
MYH: Autosomal recessive familial adenomatous polyposis
OV: Office visit
OP: Outpatient visit
OTH: Other healthcare services
RESET: the Ramsey Regression Equation Specification Error Test
RX: Prescription drugs
TOT: All costs
